# Quinolinic Acid and Nuclear Factor Erythroid 2-Related Factor 2 in Depression: Role in Neuroprogression

**DOI:** 10.3389/fphar.2019.00452

**Published:** 2019-05-21

**Authors:** Yashika Bansal, Raghunath Singh, Ishwar Parhar, Anurag Kuhad, Tomoko Soga

**Affiliations:** ^1^ Pharmacology Research Lab, University Institute of Pharmaceutical Sciences UGC-Centre of Advanced Study, Panjab University, Chandigarh, India; ^2^ Brain Research Institute Monash Sunway, Jeffrey Cheah School of Medicine and Health Sciences, Monash University Malaysia, Bandar Sunway, Malaysia

**Keywords:** oxidative stress, depression, serotonin, quinolinic acid, tryptophan, Nrf2

## Abstract

Depression is an incapacitating neuropsychiatric disorder. The serotonergic system in the brain plays an important role in the pathophysiology of depression. However, due to delayed and/or poor performance of selective serotonin reuptake inhibitors in treating depressive symptoms, the role of the serotonergic system in depression has been recently questioned further. Evidence from recent studies suggests that increased inflammation and oxidative stress may play significant roles in the pathophysiology of depression. The consequences of these factors can lead to the neuroprogression of depression, involving neurodegeneration, astrocytic apoptosis, reduced neurogenesis, reduced plasticity (neuronal and synaptic), and enhanced immunoreactivity. Specifically, increased proinflammatory cytokine levels have been shown to activate the kynurenine pathway, which causes increased production of quinolinic acid (QA, an N-Methyl-D-aspartate agonist) and decreases the synthesis of serotonin. QA exerts many deleterious effects on the brain *via* mechanisms including N-methyl-D-aspartate excitotoxicity, increased oxidative stress, astrocyte degeneration, and neuronal apoptosis. QA may also act directly as a pro-oxidant. Additionally, the nuclear translocation of antioxidant defense factors, such as nuclear factor (erythroid-derived 2)-like 2 (Nrf2), is downregulated in depression. Hence, in the present review, we discuss the role of QA in increasing oxidative stress in depression by modulating the nuclear translocation of nuclear factor (erythroid-derived 2)-like 2 and thus affecting the synthesis of antioxidant enzymes.

## Introduction

Depression is a heterogeneous mood disorder characterized by mood alterations, anhedonia, low self-esteem, social withdrawal, feelings of guilt, idiopathic pain, loss of interest in enjoyable activities, and suicidal tendencies. According to the World Health Organization, more than 300 million people currently have depression and approximately 800,000 individuals with depression commit suicide every year. Suicide due to depression is the second leading cause of death among individuals 16–25 years of age (WHO, 2018).

Various antidepressants are available for the clinical treatment of depression, including typical antidepressants, atypical antidepressants, monoamine oxidase inhibitors, and selective serotonin reuptake inhibitors. These drugs’ mechanism of action aligns well with the monoamine theory of depression. This theory states that decreased levels of serotonin in the synaptic cleft in various brain regions correspond to increased activity of the monoamine degrading enzyme and decreased synthesis of serotonin by serotonergic neurons, ultimately leading to depression (Cowen and Browning, 2015).

Although advancements in medical research have contributed to increase depression treatment options, one-third of patients do not respond to conventional drug therapies. Given this, there is a pressing need to elucidate the pathophysiology of depression so that new pathways can be explored for the investigation of novel therapeutics. Emerging evidence from various clinical and preclinical studies has revealed that oxidative stress and increased activity of immune factor cascades play significant roles in the pathophysiology of depression (Liu et al., 2015; Kohler et al., 2016). Maes et al. were the first to report that abnormal activation of the immune system and hypothalamic-pituitary-adrenal axis hyperactivity occur in depression. Additionally, decreased serotonin has been implicated in depression as a consequence of cell-mediated immune activation leading to decrease availability of plasma L-tryptophan (a precursor for serotonin synthesis). Tryptophan is a common substrate for the kynurenine pathway (KP) and serotonin synthesis pathway (methoxyindole pathway). Activation of indoleamine 2,3-dioxygenase (IDO), a rate limiting enzyme of the KP in the brain and periphery, results in decreased tryptophan availability (Maes et al., 1991, 1993; Maes, 1993). Interferon (IFN)-γ is the major inducer of IDO while tumor necrosis factor-α, interleukin (IL)-6, IFN-β, and IFN-α also activate IDO to some extent. Many studies have found a positive link between neuroinflammation and IDO expression in neurodegenerative diseases and depression (Sapko et al., 2006; Kim et al., 2012; Dobos et al., 2015).

Tryptophan metabolism *via* the KP results in the production of a neurotoxin, quinolinic acid (QA), and a neuroprotective compound, kynurenic acid (KA). KA binds to the glutamate recognition site of the N-methyl-D-aspartate (NMDA) receptor and antagonizes it, while QA binds to the glycine site of the NMDA receptor with agonistic properties. Thus, KA prevents excitotoxicity induced by NMDA overstimulation. Kynurenine is the first metabolite of the KP and is catalyzed by IDO. IDO enhances the activity of the kynureninase enzyme, which catalyzes the breakdown of kynurenine to anthranilic acid. However, kynureninase also prevents the activity of kynurenine aminotransferase (KAT), which catalyzes the formation of neuroprotective KA from kynurenine. Eventually, the breakdown of kynurenine is linked to neurotoxic QA production and reduced KA production (Réus et al., 2015).

Imbalance in the levels of QA and KA has been reported in patients with major depressive disorder (MDD). Furthermore, increased levels of QA exert neurotoxic effects in the brain of patients with depression (explained in the next section) (Myint et al., 2012). Studies conducted by three different groups have shown that QA acts as a pro-oxidant and is associated with oxidative stress (Behan et al., 1999; Santamaría et al., 1999; Rossato et al., 2002). Although QA is an NMDA receptor agonist, QA-induced oxidative stress occurs in both NMDA-dependent and independent fashion and requires further exploration (Orlando et al., 2001; Behan and Stone, 2002; Guillemin, 2012).

A key factor crucial to combat increased oxidative stress is nuclear factor (erythroid-derived 2)-like 2 (Nrf2). Nrf2 is a basic leucine zipper protein factor that acts as a master regulator of oxidative stress, maintains redox homeostasis, and provides protection against oxidative stress by transcribing various antioxidant enzymes. More specifically, studies have also shown downregulation of Nrf2 in depression and that Nrf2 activators, such as sulforaphane and its precursor glucoraphanin, exert antidepressive-like effects in depression (Martín-de-Saavedra et al., 2013; Yao et al., 2016).

In the present review, we discuss the role of QA, which might act as a pro-oxidant by impeding Nrf2 activity, an antioxidant protein implicated in clinical depression. Research on these two factors and their role in depression has led to emerging insight into the neuroprogression theory of depression and potential novel pharmacotherapeutics for its treatment.

## The KP and Glial Cells in Depression

**Figure 1:**
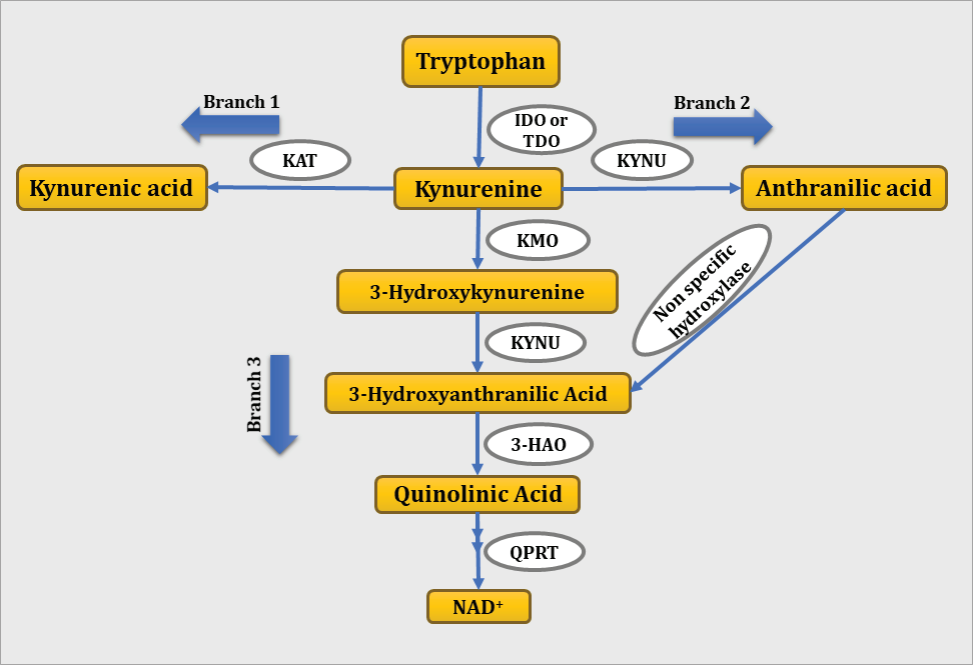
Schematic representation of tryptophan-kynurenine pathway. IDO, indoleamine 2,3-dioxygenase; TDO, tryptophan 2,3-dioxygenase; KMO, kynurenine monooxygenase; KYNU, kynureninase; 3-HAO, 3-hydroxyanthranilate 3,4-dioxygenase; QPRT: quinolinate phosphoribosyl transferase.

The KP is a metabolic pathway of tryptophan both in the periphery and central nervous system (CNS). In the periphery, 90% of tryptophan is found in the unbound form while 10% is bound to albumin. Only the free form of tryptophan can be transported through the blood-brain barrier (Jones et al., 2013). Tryptophan is metabolized to kynurenine by tryptophan 2,3-dioxygenase or IDO, a rate limiting enzyme of the KP. Tryptophan 2,3-dioxygenase catalyzes tryptophan catabolism in the liver and contributes to the peripheral levels of tryptophan, whereas IDO catalyzes tryptophan metabolism extrahepatically. In inflammatory conditions, IDO is induced by proinflammatory cytokines and shifts tryptophan metabolism to kynurenine. Kynurenine is further metabolized *via* three branches to KA, anthranilic acid, and QA by the enzymatic activity of KAT, kynureninase, and kynurenine monooxygenase, respectively. As shown in Figure 1, kynurenine is metabolized to KA through branch 1, to anthranilic acid through branch 2, and to 3-hydroxy kynurenine through branch 3. 3-Hydroxykynurenine is further metabolized to 3-hydroxyanthranilic acid in the presence of kynureninase. Finally, 3-hydroxyanthranilic acid is metabolized to QA in the presence of 3-hydroxyanthranilate 3,4-dioxygenase. Anthranilic acid formed *via* branch 2 is readily metabolized to 3-hydrocyanthranilic acid through non-specific hydroxylase, which further contributes to the synthesis of QA (Lima, 1998).

**Figure 2:**
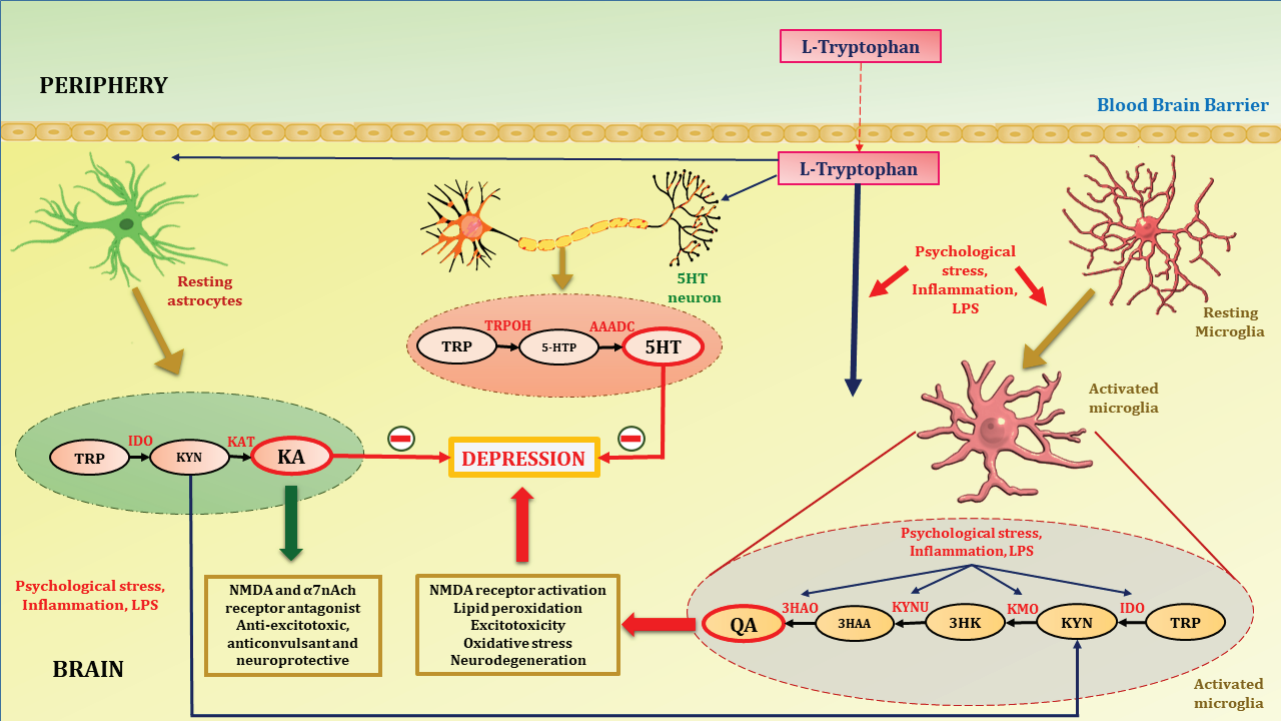
Tryptophan metabolism in astrocytes, microglia and 5HT neuron. Tryptophan, precursor of serotonin is transported to brain with the aid of non-specific competitive L-type amino acid transporters. At homeostatic conditions tryptophan is metabolized to KA in astrocytes and 5HT in serotonergic neurons. KA is an NMDA and α7nAch receptor antagonist, hence acts as anti-excitotoxic and anticonvulsant, thus provide neuroprotection. Increased activation of inflammatory cascades either by psychological stress and LPS activates microglia (microgliosis). Increased inflammation due to psychological stress or LPS activates the enzymes of neurotoxic branch of kynurenine pathway in microglia. This leads to increased production of QA. Psychological stress and activation of inflammatory cascades diverts the metabolism of tryptophan towards QA. This shift hampers the neuroprotection provided by KA and decreases synthesis of 5HT in serotonergic neurons thus contribute to the depression pathophysiology. TRP, tryptophan; IDO, indoleamine-2,3-dioxygenase; KAT, kynurenine aminotransferase; TRPOH, tryptophan hydroxylase; AAADC, aromatic L-amino acid decarboxylase; 5HT, serotonin; 5HTP, 5-hydroxytryptophan; QA, quinolinic acid; KA, kynurenic acid; KYN, kynurenine; 3HK, 3-hydroxykynurenine; 3HAA, 3-hydroxyanthranilic acid; KMO, kynurenine monooxygenase; KYNU, kynureninase; 3HAO, 3-hydroxyanthranilate 3,4-dioxygenase.

Glial cells, i.e., astrocytes and microglia, play a significant role in the development and proper function of the adult brain. Astrocytes are crucial for the formation and maturation of synapses, receptor trafficking, control of the homeostasis of ions and energy metabolites, and clearance of neurotransmitters for maintenance of the neuronal microenvironment (Araque et al., 2014; Dallérac and Rouach, 2016). Astrocytes and microglia have been found to play a potential role in inflammatory and neurodegenerative diseases as they act as both source and target of various inflammatory cytokines. Increased astrogliosis and microgliosis have been observed in several neurological and neurodegenerative disorders such as CNS injury, brain tumors, Huntington’s disease, stroke, epilepsy, Parkinson’s disease, or Alzheimer’s disease, whereas no astrogliosis takes place in depression. Postmortem studies have revealed considerably reduced number and packing density of astrocytes in subjects with depression compared to age-matched normal controls (Cotter et al., 2002; Gittins and Harrison, 2011). In addition, immunohistochemical analysis of glial fibrillary acidic protein, an astrocytic marker, revealed a significantly reduced area covered by glial fibrillary acidic protein-positive cell bodies and processes in various brain regions of young patients with depression compared to a control group (Müller et al., 2001; Fatemi et al., 2004; Miguel-Hidalgo et al., 2010; Gittins and Harrison, 2011; Chandley et al., 2013). In a recent preclinical study by Mendoza et al. (2018), reduced number and complexity of astrocytes in the dentate gyrus region of the hippocampus was seen in a restraint stress mouse model of depression. Contrary to this, animal studies have shown a clear increase in microglial activity in depressed rodents. Mice subjected to lipopolysaccharide (LPS)-induced microglial activation showed depressive-like behaviors (Henry et al., 2008). In a clinical study by Setiawan et al. (2015), significant elevation in the brain translocator protein density, a marker of microglial activation and neuroinflammation, was found in the prefrontal cortex, anterior angulate cortex, thalamus, hippocampus, dorsal putamen, and ventral striatum in patients with MDD compared to healthy controls. The mechanism by which activated microglia induce depression is through activation of microglial IDO and a further signaling pathway, i.e., the KP. Psychological stress, increased glucocorticoid levels, increased inflammatory cytokines, mainly IFN-γ but also tumor necrosis factor-α, IL-6, and their inducers such as LPS, activate microglial IDO (Kiank et al., 2010; Dantzer et al., 2011; Vaváková et al., 2015). Microglial KP-mediated depression is supported by clinical studies where IFN-α-induced immunotherapy increased peripheral and central KP metabolites. The severity of depressive-like symptoms is positively correlated with increased tryptophan metabolism (Capuron et al., 2003; Raison et al., 2010; Savitz, 2017). Additionally, postmortem studies of patients with unipolar depression have shown elevated number of QA-positive microglia in the subgenual anterior cingulate gyrus and anterior mid-cingulate cortex (Steiner et al., 2011). 1-Methlytryptophan (an inhibitor of IDO) and ketamine (an NMDA antagonist) significantly decreased depressive-like behavior by inhibiting QA-mediated NMDA signaling in rodents in an LPS-induced depression model (Aan Het Rot et al., 2012; Dobos et al., 2015). These findings revealed the prominent role of the KP in astrocytes and microglia in depression (Figure 2).

## Quinolinic Acid in Depression

QA is a neurotoxic compound involved in the neuroprogression of depression. Neuroprogression is a term inclusive of the various stages of neurodegeneration including apoptosis, reduced neurogenesis, reduced plasticity (neuronal and synaptic), and increased immunoreactivity (Berk et al., 2011). Various dynamics are involved in the neuroprogression of depression, including disturbances in the serotonergic system, increased inflammation and cell-mediated immunity, oxidative and nitrosative stress, and neurotoxic compound production (Vaváková et al., 2015; Ruiz et al., 2018). QA is one such neurotoxic compound that acts as an NMDA receptor agonist and elicits excitotoxic damage *via* glutamatergic activation of neurons and astrocytes (Zhou et al., 2013).

The serotonin theory of depression asserts that “depression is a mood disorder characterized by decreased levels of monoamines, i.e., serotonin in the brain as a consequence of the increased activity of monoamine degrading enzyme, i.e., MAO or reduced serotonin synthesis” (Delgado, 2000). The reduced synthesis of serotonin accounts for the activation of the KP, as a consequence of increased inflammatory cascades in the brain (Ball et al., 2007). Critically, the fate of tryptophan depends on proinflammatory factor levels in the brain. During stress, the levels of circulating and brain proinflammatory cytokines (IL-6, IL-β, tumor necrosis factor-α, and IFN-γ) increase (Lanquillon et al., 2000; Kaestner et al., 2005; Thomas et al., 2005). This increase activates IDO and thus shifts tryptophan metabolism toward the tryptophan-KP (Leonard, 2010). Under basal conditions, kynurenine is metabolized predominantly to KA in astrocytes, the glia cells responsible for maintenance of homeostasis within the brain. KAT catalyzes the synthesis of KA from kynurenine and is not responsive to increased inflammation as are other enzymes of the KP. KAT is predominantly expressed in astrocytes (Rossi et al., 2008). During neuroinflammation, kynurenine metabolism shifts toward QA synthesis in microglia due to microglial activation (Delgado, 2000). Increased kynurenine levels and kynurenine monooxygenase expression are associated with neuroinflammation, characterized by activation of microglia and inflammatory cascades in the CNS. This shift from the methoxyindole pathway toward the KP increases the levels of the deleterious metabolite, QA, in the brain during increased neuroinflammation (Yan et al., 2015).

IDO-directed activity of pro-inflammatory cytokines (IL-6, IFN-α, and IFN-γ) can outpace KA synthesis by inhibiting KAT. A clinical study by Savitz et al. revealed reduced KA/QA levels in the cerebrospinal fluid (CSF) of patients with depression, MDD, and remitted patients with MDD compared to healthy individuals (Savitz et al., 2015). Additional studies have also shown a negative correlation between hippocampal and amygdala volume and increased QA levels in patients with bipolar disorder (Savitz et al., 2015). Another study reported that QA and IL-6 levels in the CSF are positively correlated with suicide attempts in patients with MDD. Clinical and postmortem studies with individuals who had attempted to commit suicide have shown clear increase in the levels of QA in the brain (Erhardt et al., 2013; Brundin et al., 2016). In a preclinical study by Laugeray et al. (2011), tryptophan was found to be metabolized to QA in the amygdala and striatum but not in the cingulate cortex. Furthermore, peripheral catabolism of tryptophan to kynurenine was found to increase in a murine model of depression, resulting in lower availability of plasma tryptophan to the brain (Laugeray et al., 2011).

Given the relationship between QA and depression, the next question that arises is how QA serves as an oxidative stress modulator. QA is not only excitotoxic but also a modulator of oxidative stress (Kubicova et al., 2013). Specifically, QA increases reactive oxygen species (ROS) by increasing the excitotoxicity of the NMDA receptor (Guillemin, 2012; Schwarcz et al., 2012). QA-induced toxicity can be mediated through both NMDA-dependent and independent mechanisms. In line with this, studies have shown that at pathological concentrations (i.e., 10–40 μM), QA forms complexes with iron (Fe) *via* the Fenton reaction and thus increases ROS levels (Stipek et al., 1997; Iwahashi et al., 1999; Müller et al., 2007; Kubicova et al., 2013). In a study by Goda et al. (1996), it was found that the QA-Fe^2+^ complex causes cell death *via* hydroxyl ion-induced DNA chain breakage and lipid peroxidation. QA also enhances free radical production by inducing nitric oxide synthase activity in neurons and astrocytes and also increases poly(ADP-ribose) polymerase and extracellular lactate dehydrogenase activities and impairs mitochondrial function (Braidy et al., 2010; Pérez-De La Cruz et al., 2010). QA has also been found to impair the activity of endogenous antioxidant enzymes. In a study by Rodríguez-Martínez et al. (2000), intrastriatal injection of QA reduced the activity of reduced glutathione and cytosolic copper/zinc superoxide dismutase, whereas it increased the levels of oxidized glutathione. Melatonin and deprenyl, potent free radical scavengers, prevented QA-induced oxidative stress *via* NMDA receptor-independent action and also elevated antioxidant enzyme levels (Behan et al., 1999; Antunes Wilhelm et al., 2013). Increased brain microglial density was reported by a postmortem study with individuals who had attempted to commit suicide (Steiner et al., 2008). In addition, microglial QA levels have also been shown to be upregulated in the brain of patients with depression (Steiner et al., 2011). In their study with patients with depression, Meier et al. found a positive correlation between a decreased KA/QA ratio in the CSF and reductions in gray matter volume in the rostral and subgenual anterior cingulate cortex in the medial prefrontal cortex (Meier et al., 2016). Patients with bipolar disorder were found to have decreased KA levels in the hippocampus and amygdala compared with healthy controls (Savitz et al., 2015). A recent meta-analysis by Ogyu et al. (2018) revealed increased QA levels and decreased KA and kynurenine levels in patients with depression. Additionally, at pathological concentrations (i.e., 10–40 μM), QA reduces metabolic and physical buffering of neurons by instigating astrocytic apoptosis and thus hampering protection to neurons from ROS and inflammatory processes that lead to neurodegeneration (Grant and Kapoor, 1998). Given this, constraining the activity of QA may reduce the neuroprogression of depression and thus serve as a potential target for its pharmacotherapeutic treatment.

## Nrf2 in Depression

**Figure 3:**
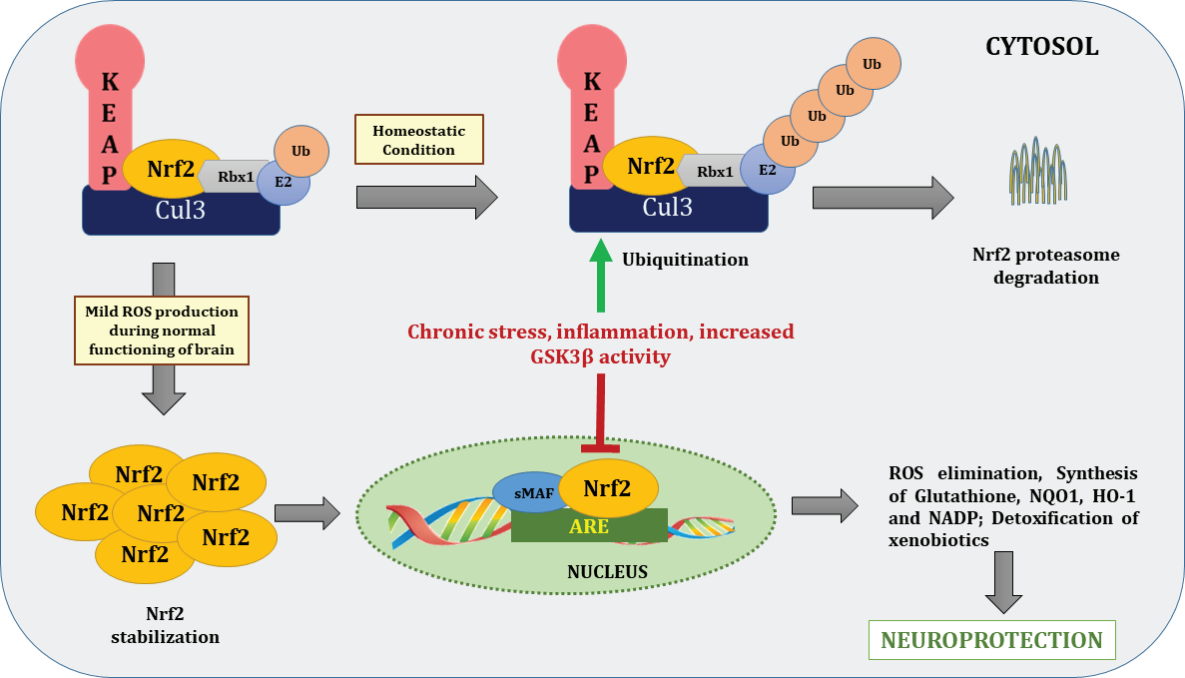
Nrf2/ARE pathway during basal and stressed conditions. During homeostatic conditions Nrf2 remains in tethering with Keap1 and Cul3-Rbx1-E2 ligase and undergoes proteasome degradation through ubiquitination. In normal brain functioning, mild ROS production dissociates Nrf2 from Keap1 and translocate it to the nucleus. After nuclear translocation, it binds with ARE to transcribe genes of various antioxidants enzymes to combat deleterious effects of ROS. On contrary during chronic stress, increased activity of inflammatory cascades and GSK-3β increased Nrf2 proteasome degradation via ubiquitination. Nrf2, nuclear factor (erythroid-derived 2)-like 2; Keap1, Kelch-like ECH associated protein-1; Cul-3, cullin 3; ROS, reactive oxygen species; ARE, antioxidant response element; Rbx1, ring box-1; E2, E2 ubiquitin-conjugating enzyme; Ub, ubiquitin.

Nrf2, also known as NFE2L2, is a redox-sensitive transcription factor. It is a basic leucine zipper protein factor that belongs to the cap “n” collar subfamily. It plays an important role in the body’s endogenous antioxidant defense system by maintaining the intracellular redox homeostasis that when dysregulated, triggers oxidative stress (Moi et al., 1994). Nrf2 transcribes various antioxidant enzymes to protect against oxidative stress induced by injury and/or inflammation. Under physiological conditions, Nrf2 is retained in the cytosol in a dormant form *via* tethering to Kelch-like ECH associated protein-1 (Keap-1), an adaptor for the cullin-3/Rbx complex. Keap-1, in association with cullin-3/Rbx, causes ubiquitination and finally causes proteasomal degradation of Nrf2. Given this, Nrf2 is highly unstable at physiological conditions due to its negative regulation by Keap-1 (Dhakshinamoorthy and Jaiswal, 2001). In inflammatory and oxidative stress conditions, Nrf2 translocates to the nucleus after dissociating from Keap-1 and binds to antioxidant response element, also referred to as electrophile responsive element. Transcription of electrophile responsive element target genes that encode phase-II antioxidant proteins such as GCLC, NOQ1, and HO-1; detoxifying enzymes, antiapoptotic proteins, and proteasomes are then enhanced (Figure 3; Niture et al., 2014).

Of special relevance here, studies have shown that downregulation of Nrf2 occurs in depression. In a recent study by Bouvier et al. (2016), rats that were exposed to persistently stressful conditions became progressively more vulnerable to depression due to persistent oxidative stress. This oxidative stress was found to be caused by diminished nuclear translocation of Nrf2, which prevented the transcription of antioxidants and detoxifying enzymes. In an *in vitro* study by Rojo et al. (2008), GSK-3β downregulated Nrf2 in neuronal cultures, providing a possible mechanism of inability to combat oxidative stress under persistent oxidant exposure (Figure 3).

The neurotrophic hypothesis of depression proposes that depression is also accompanied by decreased neurotrophic support, which is primarily linked to the brain-derived neurotrophic factor (BDNF) protein. A recent study reported that BDNF-deficient mice were more susceptible to stress-induced oxidative damage, indicating a direct link between oxidative stress and BDNF levels (Hacioglu et al., 2016). Bouvier et al. (2016) reported a positive correlation between BDNF and Nrf2 levels in rodents vulnerable to depression. In a study by Mendez-David et al. (2015), a positive correlation between BDNF and Nrf2 in the hippocampus of corticosterone-treated depressed rats was established. Besides depression, reduced levels of Nrf2 have further been noted in hippocampal astrocytes in patients with Alzheimer’s disease (Ramsey et al., 2007). Furthermore, when Nrf2 knockout mice were treated with corticosterone, they exhibited significant changes in prefrontal cortex neurotransmitter levels, including serotonin, glutamate, and dopamine, compared to wild-type controls (Mendez-David et al., 2015).

In addition to its role in neurotransmission and depression, Nrf2 is also linked to neuroprotection. Nrf2 protects neurons from the deleterious effects of oxidative and nitrosative stress and inflammatory cytokines by regulating astrocytic enzymes integral to glutathione redox signaling (Habas et al., 2013; Baxter and Hardingham, 2016). In a recent study by Yao et al. (2016), mice exhibiting depression-like behavior were found to have decreased levels of Keap-1 and Nrf2 protein in the prefrontal cortex, CA3, and dentate gyrus compared to controls. The authors also found that pre-treatment with dietary sulforaphane and 0.1% glucoraphanin (Nrf2 modulators) during the juvenile and adolescent stages prevented emergence of depression-like behavioral phenotypes in animals exposed to repeated social defeat stress (Yao et al., 2016). Additionally, Freitas et al. (2016) found that agmatine, an endogenous metabolite of L-arginine, protects corticosterone-induced apoptotic cell death and ROS production in hippocampal neuronal cells *via* induction of Nrf2. This group further evaluated the antidepressant potential of agmatine in a corticosterone mouse model of depression. Agmatine showed antidepressant properties in Nrf2^(+/+)^ mice *via* induction of Nrf2 and BDNF and was unable to reverse the depression-like effect in Nrf2 knockout (Nrf2^(−/−)^) mice. Agmatine also prevented morphological changes in astrocytes and microglia in the CA1 region of the hippocampus of Nrf2^(−/−)^ mice (Freitas et al., 2016).

Nrf2 is predominantly expressed in astrocytes. In studies by Calkins et al. (2004, 2010), specific Nrf2 activation in astrocytes provided protection against malonate and 3-nitropropionic acid induced mitochondrial complex II inhibition-mediated neurotoxicity both *in vitro* and *in vivo*. Additionally, the presence of *tert*-butyl hydroquinone (tBHQ; an Nrf2 activator) was unable to buffer against oxidative stress in pure neuronal cultures, whereas in a mixed culture of astrocytes and neurons, tBHQ caused Nrf2 activation and provided protection from oxidative stress (Bell et al., 2015). In another complimentary study conducted by (Kraft, 2004), similar *in vitro* results were reported wherein the pharmacological activation of mixed cultures by tBHQ protected against oxidative stress, whereas specific inhibition of Nrf2 in astrocytes reduced the protection. Although Nrf2 is predominantly expressed in astrocytes, overactivation of Nrf2 in astrocytes is protective in various neurogenerative diseases such as Parkinson’s disease (Chen et al., 2009), amyotrophic lateral sclerosis (Vargas et al., 2008), Huntington’s disease (Shih et al., 2005), and multiple sclerosis (Draheim et al., 2016). In another study, overactivation of neuronal Nrf2 was found to be protective in Alzheimer’s disease (Vargas et al., 2013), suggesting potential neural involvement. Collectively, these findings indicate that astrocytic activation of Nrf2 may be a potent pharmacotherapeutic target in various CNS diseases.

New findings on the role of Nrf2 in various CNS diseases have shown that Nrf2 is also important in the regulation of neuroinflammation that arises as a consequence of oxidative stress in the brain. In a study by Martín-de-Saavedra et al. (2013), Nrf2 was shown to reverse depression symptoms *via* an anti-inflammatory mechanism. This study also found that depletion of Nrf2 caused inflammation-induced depressive symptoms that were reversed by treatment with an anti-inflammatory drug, rofecoxib. Furthermore, this study also found that sulforaphane treatment in an LPS-induced inflammatory mouse model of depression increased nuclear translocation of Nrf2 and led to antidepressant-like effects (Martín-de-Saavedra et al., 2013). Also in the same study, Nrf2^(−/−)^ animals displayed a reduction in BDNF expression in the hippocampus, demonstrating a prospective relationship between the neurotropic factor and Nrf2. Suggesting a potential mechanism for these effects of Nrf2, in a recent study by Kobayashi et al. (2016), Nrf2 was found to be an upstream regulator of cytokine production *via* inhibiting transcriptional upregulation of proinflammatory cytokine genes and thus decreased the levels of proinflammatory cytokines. Thus, Nrf2 exerts anti-inflammatory and antioxidant effects on biological systems. Collectively, the studies discussed here strongly suggest an essential role for Nrf2 in depression, pointing at the induction of Nrf2 as a potential target in the investigation of novel antidepressant drugs.

## Interactions Between QA and Nrf2 in Depression

As suggested by the studies outlined previously, both QA and Nrf2 play substantial, and probably opposite, roles in the etiology of depression. While QA has been shown to contribute to depression, Nrf2 likely protects against depression. Given this, it is important to understand whether QA and Nrf2 are directly associated. To date, only two known *in vitro* studies have examined the direct relationship between QA and Nrf2 (Tasset et al., 2010; Colín-González et al., 2014). Suggesting a potential mechanism for this, Tasset et al. (2010) reported that exposure to QA abolished nuclear translocation of Nrf2 in rat striatal tissues. Nuclear translocation of Nrf2 in QA-treated striatal slices was noted after tBHQ (a Nrf2 modulator) treatment (Tasset et al., 2010). This indicates that QA abolishes Nrf2 translocation in the nucleus.

An additional study by Colín-González et al. (2014) reported that striatal slices from Nrf2^(−/−)^ animals were more vulnerable to the oxidative damage caused by QA than were wild-type tissues. In mouse striatum slices, QA treatment after 1 h was found to stimulate Nrf2 nuclear translocation, while after 3 h, it was found to decrease Nrf2 translocation and phase II enzymatic activity and increase lipid peroxidation. Enhanced Nrf2 nuclear translocation may thus represent a compensatory mechanism for QA-induced oxidative stress, while downregulation of Nrf2 might contribute to cellular oxidative damage. Further, Nrf2^(−/−)^ animals were found to be less responsive to QA-induced toxicity compared to wild-type animals (Colín-González et al., 2014). In a recent study by Ferreira et al. (2018), KA prevented QA-induced oxidative imbalance, mitochondrial dysfunction, and decreased Nrf2 levels in striatal slices. The NADPH inhibitor, apocynin, prevented QA-induced decrease in antioxidant levels such as glutathione, γ-GCL, and GPx activities and Nrf2 mRNA levels which is involved in the maintenance of antioxidant levels (Cruz-Álvarez et al., 2017).

**Figure 4:**
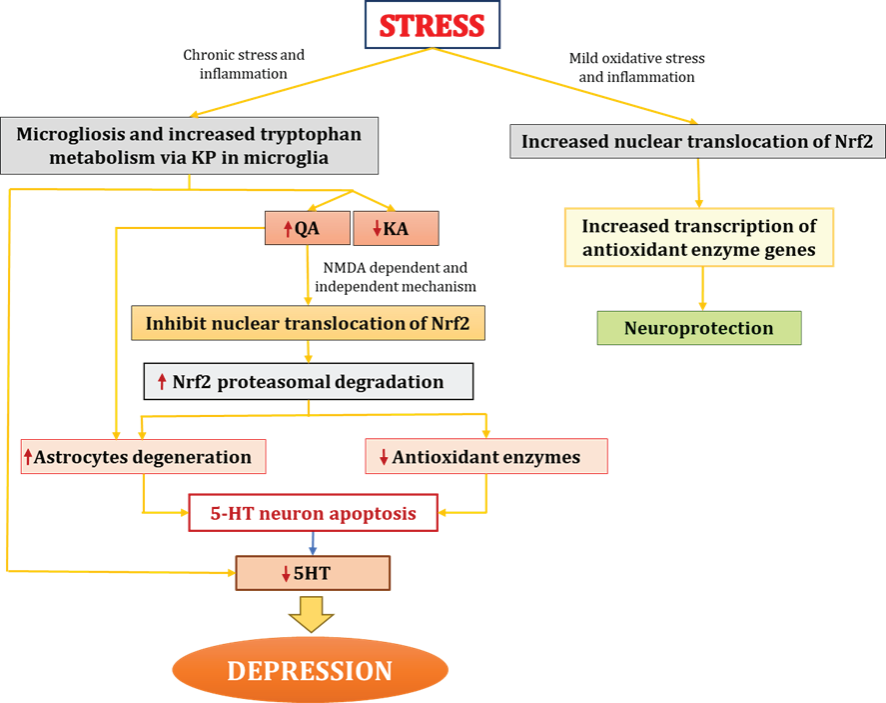
Interaction between QA and Nrf2 in depression. In chronic stress conditions, increased proinflammatory cytokines lead to microgliosis. Increased proinflammatory cytokines activate IDO (enzyme catalyzing first rate-limiting step of KP) and shift tryptophan metabolism from serotonin synthesis to KP in microglia. Elevated levels of QA further increases oxidative stress through NMDA agonistic activity and secondly might be through directly inhibiting nuclear translocation of Nrf2 and hence causes increased proteasome degradation of Nrf2 which ultimately led to decreased antioxidant levels and increased oxidative stress. QA also causes degeneration of astrocytes thus hampering protection and nutritional support to neurons. All this together lead to degeneration of 5HT neurons and decreased 5HT synthesis which results in depression. On the other hand mild oxidative stress stabilizes Nrf2 and increases Nrf2 transcribed antioxidant enzymes. IDO, indoleamine-2,3-dioxygenase; KP, kynurenine pathway; QA, quinolinic acid; KA, kynurenic acid; Nrf2, nuclear factor (erythroid-derived 2)-like 2; 5HT, serotonin.

Apart from NMDA-induced excitotoxicity by QA, studies have also shown that QA exerts toxic effects by increasing the oxidant-to-antioxidant ratio. Specifically, QA alters the ratio of reduced glutathione to oxidized glutathione and hinders the activity of other antioxidants, such as Cu and zinc-superoxide dismutase (Rodríguez-Martínez et al., 2000). However, Nrf2 protects against these pro-oxidant effects by increasing the transcription of various cytoprotective antioxidant genes such as *GSH*. For example, Harvey et al. (2009) demonstrated that Nrf2 is protective against oxidative stress by maintaining the glutathione redox state *via* transcriptional regulation of glutathione reductase. As noted previously, pathological concentrations of QA can cause astrocytic apoptosis, limiting the role of these cells in protecting neurons from ROS and other pro-inflammatory processes, thus causing neurodegeneration (Grant and Kapoor, 1998). In sum, Nrf2 protects against the deleterious effects of oxidative and nitrosative stress and inflammatory cytokines on neurons by cytoprotection of astrocytes *via* regulation of enzymes belonging to the glutathione redox system (Habas et al., 2013; Baxter and Hardingham, 2016). Based on the aforementioned studies, there may also be a direct link between QA and Nrf2, wherein QA constrains Nrf2 nuclear translocation. Further examining this process may serve as a viable potential target for the development of pharmacological treatments for depression (Figure 4).

## Concluding Remarks

A growing body of preclinical and postmortem evidence has revealed that depression is often accompanied by hippocampal and prefrontal cortex neuronal atrophy. Decreased neuronal activity and plasticity across multiple brain regions have also been noted in clinical and modeled depression. Collectively, these morphological changes and altered neuronal functions may be due to increased oxidative stress, which contributes to neuronal and glial atrophy in depression. Specifically, the role of QA in mediating these changes and the broader pathophysiology of depression has become a subject of clinical and preclinical studies. A decreased KA/QA ratio has been noted in the CSF of remittent patients with depression. Moreover, QA is an endogenous toxin that also acts as a pro-oxidant and leads to the neuroprogression of depression. Furthermore, QA is linked to the activity of Nrf2, an endogenous antioxidant transcription factor. While the contributions of Nrf2 to CNS diseases have been understudied, it has been reported that Nrf2 is both a potent antioxidant transcription factor and also has anti-inflammatory effects that may be responsible for its antidepressant properties. Hence, further study of the associations among QA, Nrf2, and oxidative stress may provide novel insight into the neuroprogression and etiology of depression.

## Author Contributions

YB wrote this review. RS and AK helped editing a draft review. IP and TS designed the flow of review paper, contributed to English editing and made scientific comments.

### Conflict of Interest Statement

The authors declare that the research was conducted in the absence of any commercial or financial relationships that could be construed as a potential conflict of interest.
